# Gender Differences after Digital Interventions in the Golden Hours after Traumatic Events

**DOI:** 10.1192/j.eurpsy.2022.1739

**Published:** 2022-09-01

**Authors:** S. Lahutina

**Affiliations:** Bogomolets National Medical University, Medical Psychology, Psychosomatic Medicine And Psychotherapy, Kyiv, Ukraine

**Keywords:** Stress, Gender differences, traumatic event, digital technology

## Abstract

**Introduction:**

Digital technologies are used in the prevention of post-traumatic stress disorder (PTSD). There is no clear evidence for effective gender-sensitive preventive measures for PTSD. Using Tetris during the golden hour after trauma can reduce intrusive memories and thus reduce the likelihood of PTSD in the future.

**Objectives:**

Understand the features of gender differences after psychological interventions in patients in the acute period after a traumatic event. Video games that use visual-spatial efforts over a fixed time and frequency (Tetris) may reduce the likelihood of developing PTSD.

**Methods:**

Main inclusion criteria was an exposure to traumatic event (time from traumatic event - 0-24 hours). Respondents were assessed by PTSD symptom scale (PCL-5), peritraumatic distress scale (PDS), peritraumatic dissociative experience scale (PDES) and global functioning scale (GFS), intrusion diary (intervals: week 0, week 4, week 8, week 12).

**Results:**

PTSD symptoms were more severe in female participants (p ≤ 0,05). Participants in the Tetris game group recorded significantly fewer intrusive memories during the first week after the traumatic event than participants in two other groups, with a mean effect size of 57 (M = 8.73 vs. M = 23.26, t (69) = 2.80, P = 0.005, d = 0.67, 95% CI: 0.18, 1, 14). After the first month of follow-up, members of the Tetris game group reported less stress from intrusive symptoms.

**Conclusions:**

Tetris intervention may reduce intrusive memories of real trauma. Women had more severe PTSD symptoms. Due to the small number of samples, the study should be repeated.

**Disclosure:**

No significant relationships.

